# Transcriptomic and Metabolomic Analysis of the Effects of Exogenous Trehalose on Salt Tolerance in Watermelon (*Citrullus lanatus*)

**DOI:** 10.3390/cells11152338

**Published:** 2022-07-29

**Authors:** Gaopeng Yuan, Dexi Sun, Guolin An, Weihua Li, Wenjing Si, Junpu Liu, Yingchun Zhu

**Affiliations:** Zhengzhou Fruit Research Institute of the Chinese Academy of Agricultural Sciences, Zhengzhou 450000, China; yuangaopeng@caas.cn (G.Y.); sundexi@caas.cn (D.S.); anguolin@caas.cn (G.A.); liweihua@caas.cn (W.L.); siwenjingsmile@sina.cn (W.S.)

**Keywords:** transcriptomic, metabolomic, trehalose, salt tolerance, watermelon, bHLH family transcription factors

## Abstract

Trehalose can effectively protect the biomolecular structure, maintain the balance of cell metabolism, and improve the tolerance to various abiotic stresses in plants. However, the molecular mechanism underlying the improvement in salt tolerance by exogenous trehalose in watermelon (*Citrullus lanatus)* seedlings is still unclear. To understand these molecular mechanisms, in this study, watermelon seedlings under salt stress were treated with various concentrations of exogenous trehalose. An amount of 20 mM exogenous trehalose significantly improved the physiological status; increased the activities of enzymes such as POD, SOD, and CAT; and increased the K^+^/Na^+^ ratio in watermelon seedlings under salt stress. RNA-seq and metabolomic analysis were performed to identify the specifically expressed genes and metabolites after trehalose treatment. Watermelon seedlings were divided into salt stress (CK2), control (CK1) and trehalose treatment (T) groups as per the treatment. Overall, 421 shared differentially expressed genes (DEGs) were identified in the two comparison groups, namely CK2–CK1 and T–CK2. Functional annotation and enrichment analysis revealed that the DEGs were mainly involved in MAPK signaling pathway for plant hormone signal transduction and phenylpropanoid biosynthesis. Furthermore, 129 shared differential expressed metabolites (DEMs) were identified in the two comparison groups using liquid chromatography–mass spectrometry, which were mainly involved in the metabolic pathway and phenylpropanoid biosynthesis. The combined transcriptomic and metabolomic analyses revealed that genes involved in phenylpropanoid biosynthesis, plant hormone signal transduction, and carbohydrate biosynthesis pathways, especially bHLH family transcription factors, played an important role in improving salt tolerance of watermelon seedlings after exogenous trehalose treatment.

## 1. Introduction

Soil salinization is an increasingly serious global problem. Globally, at present, salinized land accounts for approximately 10% of the land area, of which 20% corresponds to cultivated land and 50% corresponds to irrigated land [1]. In China, saline–alkali land area is 1.5 × 10^9^ hm^2^, which is one of the important environmental factors limiting agricultural production. High salinity affects plants in various ways, including osmotic stress, ion imbalance and toxicity, nutritional problems, oxidative stress, metabolic changes, membrane damage, reduced cell division, and expansion [2,3]. This results in the disruption of many important metabolic functions in plant cells; it can cause serious adverse effects on the plant growth and development, even leading to death [4,5]. In response to a signal provided by a specific receptor, a specific signaling pathway is activated, inducing a cascade of secondary signals and protein phosphorylation, e.g., MAPK signaling [6]. In addition, secondary messengers such as reactive oxygen species (ROS), calcium, nitric oxide, H_2_S, H_2_O_2_, phospholipids, and plant growth regulators are involved in response to salt stress [7,8]. In the process of adaptation to salt stress, transcription levels of some genes in plants change to adapt to salt stress. Some genes and transcription factors (TFs) related to morphological structure and physiological metabolism play a key role in regulating the expression of stress-response genes. For example, *WRKY, MYB, NAC, bZIP, AP2/ERF, bHLH*, and TFs from other family act as central regulators and molecular switches in the complex signal transduction network under salt stress [9], which can further control the expression of downstream genes or directly protect plants to reduce damage due to salt stress [10,11,12].

One of the necessary conditions for plant to adapt to salty environment is its ability to maintain the balance of ions in plant cells [13]. Trehalose and its intermediate molecules can activate the necessary salt response system in plants, maintain osmotic pressure of cells, protect membrane structure, participate in signal transduction process, and effectively protect biomolecular structure of plants, thereby maintaining metabolic balance in plants [14,15,16,17]. Trehalose biosynthesis involving trehalose phosphate synthase (TPS) and trehalose phosphate phosphatase (TPP) was the most widely studied pathway in plants. In addition, trehalase also exists in this pathway, resulting in the rapid degradation of trehalose after the completion of its cellular functions, which may explain its low homeostasis in plants [18]. In higher plants, *TPS* and *TPP* are generally widespread in the form of large gene families [19]. The overexpression of *TPS* or *TPP* gene can significantly increase trehalose content in transgenic plants and improve stress tolerance [20,21,22,23,24,25,26,27].

In addition to endogenous trehalose, the exogenous application of trehalose can improve the tolerance of plants to various plant stresses, such as salt, low temperature, drought, and waterlogging. In general, exogenous trehalose promotes the tolerance of plants to stress through the following aspects: (1) improving the activities of related enzymes such as superoxide dismutase (SOD), ascorbate peroxidase (APX), peroxidase (POD), and catalase (CAT) in plants [28,29]; (2) increasing proline (Pro) content and ROS scavenging capacity and decreasing superoxide anion (O_2_^-^) formation rate and malondialdehyde (MDA) content [30]; (3) promoting intracellular K^+^ uptake and Na^+^ efflux and regulating intracellular K^+^/Na^+^ balance [31,32,33,34]; (4) inducing the expression of stress related genes and trehalose synthesis pathway genes, such as *SnRKs*, and abscisic acid (ABA) synthesis and metabolism-related genes, such as *TPS* and *TPP* [35].

Watermelon is a moderately salt-sensitive crop. When the salt content in soil reaches 0.3%, stress occurs, and plant growth is significantly inhibited, leading to a serious decline in watermelon yield and quality [36]. Therefore, it is of great significance for the safety and sustainable development of watermelon industry to explore the salt tolerance mechanism of watermelon, explore salt tolerance genes in watermelon, and then cultivate new salt tolerant watermelon varieties. In this study, watermelon seedlings under salt stress were treated with various concentrations of exogenous trehalose to obtain the optimal concentration of trehalose that can improve the salt tolerance of watermelon. Subsequently, RNA sequencing and metabolomic analysis were performed to explore the molecular mechanism of exogenous trehalose improving the salt tolerance of watermelon seedlings and to identify key candidate genes. Therefore, this study provided a theoretical basis for the breeding of salt-tolerant watermelon varieties.

## 2. Materials and Methods

### 2.1. Plant Materials and Treatments

The seeds of watermelon (‘HQ-2′ variety) were sown in clay after germination and transplanted into Hoagland solution after cotyledons were fully expanded (growth conditions: 16 h/8 h light/dark cycle). After 30 days of growth, the seedlings were cultured in Hoagland solution containing 150 mM NaCl with 0 (the CK2 group), 5, 10, 20 (the T group) and 30 mM trehalose in intelligent greenhouse (25 °C and 16 h/8 h light/dark cycle) for 7 days. Hoagland solution was changed every other day at 8:00 am. The control plants were cultured in standard Hoagland solution without NaCl and trehalose (the CK1 group). There were 10 seedlings in each treatment group with 3 biological replicates. Roots of seedlings were sampled after 7 days of treatment. Some of the samples were stored at −20 °C for physiological and biochemical index determination. The other part was equally divided into two parts, and the samples were frozen in liquid nitrogen and stored at −80 °C for RNA extraction and sequencing, and metabolomic analysis. When sampling, 10 seedlings were mixed to form one sample, and 3 biological replicates were maintained for each treatment.

### 2.2. Measurements of Physiological and Biochemical Index

The plant height (the vertical height from the basal part of stem to the top of the plant) was measured using a 30 cm (1 mm) ruler.

The stem diameter (1 cm above stem base) was measured using a vernier caliper (SHRN 0–300 mm, Guilin, China).

The whole seedling plant was weighed. First, the fresh weight (FW) of the seedling was weighed by electronic balance, and the seedling was dried in an oven until constant weight, and then dry weight (DW) was measured.

The MDA, POD, SOD, and CAT content were determined according to Yuan et al. [26] using KTB1050, KTB1150, KTB1030, and KTB1040 (Abbkine, Beijing, China) kits, respectively. The Na^+^ and K^+^ content were determined using sodium assay kit (C002-1-1) and potassium assay kit (C001-2-1) (Nanjing Jiancheng Bioengineering Institute, Nanjing, China), respectively. SpectraMax i3X Multi-mode Detection Platform Molecular Devices (Molecular Devices, Shanghai, China) was used to measure the absorbance.

### 2.3. RNA Extraction, Sequencing and Expression Profiling

Total RNA was isolated from the roots of 9 samples, with CK1, CK2, and T (20 mm trehalose treatment). RNA was extracted using RNeasy Plant Mini Kit (Tiangen, Beijing, China) according to the manufacturer’s instructions. Total RNA was purified and subsequently identified and quantified. High quality and more than 200 ng RNA was reverse transcribed to cDNA based or polyA tail. The template was switched to the 5’ end of RNA, and a full-length cDNA was generated using PCR. The PCR product was detected using Agilent 2100 (Thermo Fisher Scientific, Waltham, MA, USA). Purified cDNA from previous steps was fragmented into small pieces using fragment buffer (5× First strand buffer, Invitrogen, USA) by PCR, and the product was purified and selected by the Agencourt AMPure XP-Medium kit (Thermo Fisher Scientific, Waltham, MA, USA). The cDNA was quantitatively analyzed using Agilent 2100 bioanalyzer. The double chain PCR product was thermally denatured and cycled by splinting oligonucleotide sequences through QC steps. At last, format single strand circle DNA into the final library. The final library was phi29 (Thermo Fisher Scientific, Waltham, MA, USA) manufactured DNA nanospheres by loading more than 300 copies of a molecule’s DNA nanospheres into a patterned nanoarray. For each sample, the cDNA library was sequenced using BGISEQ-500 system (Shenzhen, China). The read length of the BGISEQ-500 system was 100 bp.

Clean reads were obtained by screening the sequencing data, subsequently mapped them to the genome of watermelon in CuGenDB (http://cucurbitgenomics.org/organism/21, accessed on 25 March 2022) using Bowtie2 (http://bowtie-bio.sourceforge.net/index.shtml, accessed on 25 March 2022). Then, clean reads were mapped to the genome sequence using Bowtie2, and gene expression levels were calculated by FPKM (fragments per kilobase million). Gene annotation and function assignment were performed based on KEGG (http://www.genome.jp/kegg/, accessed on 25 March 2022) and GO (http://www.geneontology.org/, accessed on 25 March 2022) databases. Differentially expressed genes (DEGs) were defined as fold change ≥ 2.00 and FDR ≤ 0.001. Through GO enrichment and KEGG enrichment pathway, the metabolic pathways with significant enrichment were identified and compared with the genome-wide background. DEGs were functionally classified according to GO and KEGG annotation results and official classification. Under normal circumstances, FDR ≤ 0.01 is considered significant enrichment. For plant transcription factors, we used getorf to detect the ORF (open reading frame) of unigene and then used HMM search to compare the ORF with the transcription factor protein domain (data from transcription factors). Then, the ability of unigene was then identified according to the transcription factor family characteristics described by PlantTFDB (http://planttfdb.cbi.pku.edu.cn, accessed on 25 March 2022).

### 2.4. Extraction and Quantification of Metabolites

Metabolites were extracted from watermelon root tissue, with six replicates for each treatment. Samples cultured in Hoagland solution without NaCl and trehalose were defined to MCK1, and which cultured in Hoagland solution containing 150 mM NaCl with 0 mM trehalose and containing 150 mM NaCl with 20 mM trehalose were defined to MCK2 and MT, respectively. The detailed extraction process was as follows: (1) 50 mg tissues were weighed and extracted by directly adding 800 μL of precooled extraction reagent (MeOH:H2O (70:30, *v*/*v*, precooled at −20°C)), 20 μL internal standards mix was added for quality control of sample preparation. (2) After homogenizing at 50 Hz for 5 min using TissueLyser (JXFSTPRP, China), samples were sonicated for 30 min at 4 °C and incubated at −20 °C for 1 h. (3) The samples were further centrifuged for 15 min at 14,000 rpm, 4 °C. (4) Next, 600 μL of the supernatants were filtered through 0.22 μm microfilters and transferred to autosampler vials for LC-MS analysis.

A quality control (QC) sample was prepared by pooling 20 μL supernatant of each sample to evaluate the reproducibility and stability of the whole LC-MS analysis. The sample analysis was performed on a Waters ACQUITY UPLC 2D (Waters, Milford, MA, USA), coupled to a Q-Exactive mass spectrometer (Thermo Fisher Scientific, Waltham, MA, USA) with a heated electrospray ionization (HESI) source. Chromatographic separation was performed on a Hypersil GOLD aQ column (2.1 × 100 mm, 1.9 μm, Thermo Fisher Scientific, Waltham, MA, USA), with mobile phase A consisting of 0.1% formic acid in water and mobile phase B consisting of 0.1 formic acid in acetonitrile.

The mass spectrometric settings for positive/negative ionization modes were as follows: spray voltage, 3.8/−3.2 kV; sheath gas flow rate, 40 arbitrary units (arb); aux gas flow rate, 10 arb; aux gas heater temperature, 350 °C; capillary temperature, 320 °C. The full scan range was 100–1500 m/z with a resolution of 70,000, and the automatic gain control (AGC) target for MS acquisitions was set to 1e6 with a maximum ion injection time of 100 ms. Top 3 precursors were selected for subsequent MSMS fragmentation with a maximum ion injection time of 50 ms and resolution of 30,000, the AGC was 2e5. The stepped normalized collision energy was set to 20, 40, and 60 eV. High-resolution mass spectrometers (Q Exactive, Thermo Fisher Scientific, Waltham, MA, USA) were used to collect data in positive and negative ion modes to improve chemical composition coverage. The original mass spectrometry data collected by LC-MS/MS were imported into Compound Discoverer 3.1 (Thermo Fisher Scientific, Waltham, MA, USA) for data processing. It mainly includes peak extraction, correction of retention time within and between groups, incorporation of added ions, filling of missing values, background peak labeling and metabolite identification, etc. The molecular weight, retention time, peak area, and identification results of the compound were given.

Screening conditions for differential metabolites: (1) VIP≥1 of the first two principal components of PLS-DA model, (2) fold change ≥ 1.2 or ≤ 0.83, and (3) q-value < 0.05. Then, metabolic pathways were constructed based on the information in KEGG database.

### 2.5. Combined Transcriptomic and Metabolomic Analysis

First, the mean value of six biological duplications in the metabolite data and the normalized mean value of each gene in the transcriptomic data were evaluated. Then, the DEGs and DEMs data were converted to log2 values prior to analysis. We used Pearson correlation coefficients (PCCs) and corresponding *p*-values (PCCPs) to screen DEMs and related DEGs in combined metabolomics and transcriptomic analysis. The screening criteria were PCC > 0.90 and PCCP < 0.05 according to Cho et al. [37]. To better understand the relationship between DEGs and DEMs, we mapped DEGs and metabolites between the same treatments (CK1, CK2, and T) to their associated KEGG pathways.

### 2.6. Quantitative Real-Time PCR Validation

The first strand of cDNA was obtained from total RNA using a PrimeScript RT reagent Kit (TaKaRa, Dalian, China). Online software (https://www.ncbi.nlm.nih.gov/tools/primer-blast/index.cgi?LINK_LOC=BlastHome, accessed on 10 April 2022) was used to design specific primers, which were shown in Table S1. qPCR was performed on the Light Cycler480 Real-Time System (Bio-Rad Laboratories, Hercules, CA, USA) with the method described previously [26]: 95 °C for 5 min, 40 cycles of 95 °C for 10 s, 58 °C for 10 s, and 72 °C for 10 s. Each reaction mixture (final volume was 20 μL) contained 1.0 μL cDNA, 10.0 μL AMP SYBR Green (Low ROX) (Bioman, Chian), 1.0 μL primer (10 mM working solution) and 7.0 μL sterile water. The 2^−ΔΔCt^ method was used to calculate the expression of DEGs [38].

### 2.7. Statistical Analysis of Data

All data were statistically analyzed using office 2016 software, and SPSS 18.0 software was used to sort out the data for one-way ANOVA statistical analysis, and the significant difference was defined as *p* < 0.05 (n = 3).

## 3. Results

### 3.1. Trehalose Promoted the Growth of Watermelon Seedlings under Salt Stress

**Figure 1:**
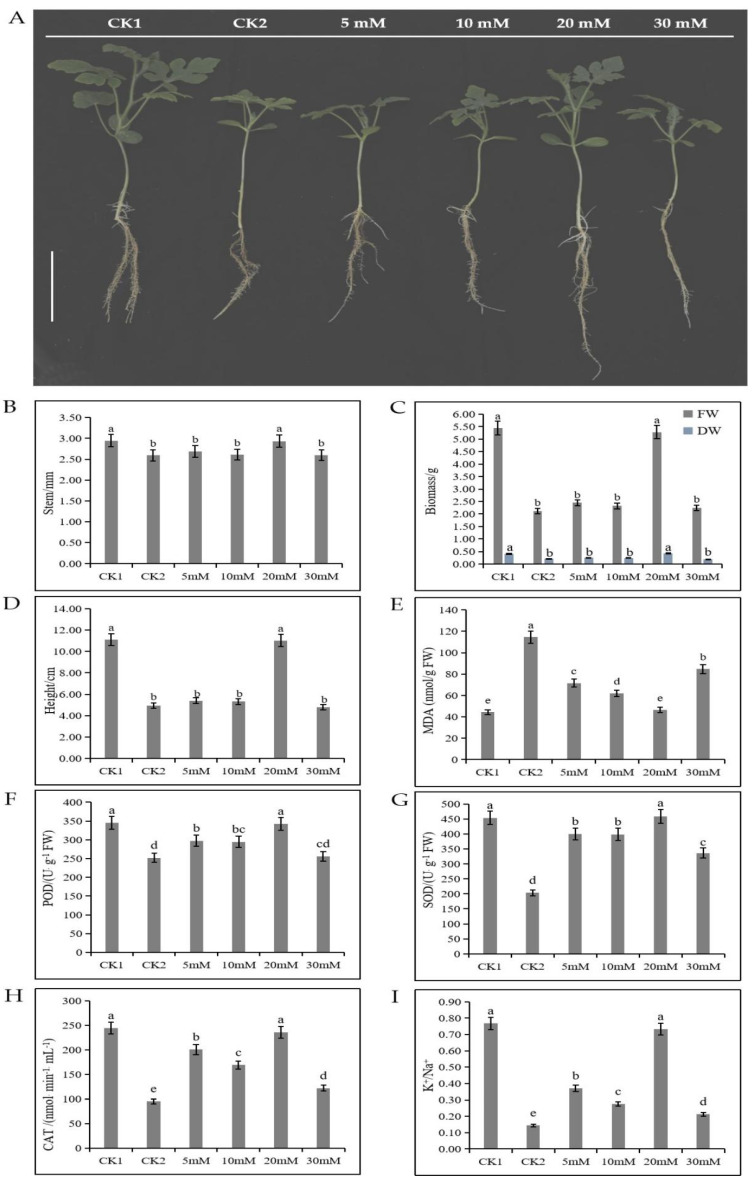
The effect of trehalose treatment on the growth of watermelon seedlings under 150 mM NaCl stress. (**A**) Phenotype of watermelon seedlings in Hoagland solution under trehalose and NaCl treatment for 1 week. Scale bar = 5 cm. (**B**) Measurements of the stem diameter, (**C**) biomass, and (**D**) plant height of seedlings. (**E**) MDA content, (**F**) POD activity, (**G**) SOD activity, (**H**) CAT activity, and (**I**) K^+^/Na^+^ ratio of roots. Error bars indicate SE (n = 3). Small letters indicate significant differences among the different treatments (*p* < 0.05). CK1 represents watermelon seedlings cultured in normal Hoagland solution; CK2 represents watermelon seedlings cultured in Hoagland solution containing 150 mM NaCl; 5 mM, 10 mM, 20 mM and 30 mM represent watermelon seedlings cultured in Hoagland solution containing 150 mM NaCl and indicated concentrations of trehalose.

To study the effect of exogenous trehalose on watermelon seedlings under salt stress, watermelon seedlings under 150 mM NaCl stress were treated with various concentrations of exogenous trehalose (5, 10, 20, and 30 mM; Figure 1). Salt stress (the CK2 group) significantly inhibited the growth of watermelon seedlings compared with the control (the CK1 group). The growth trend of watermelon seedlings under salt stress and treated with 5, 10, and 30 mM trehalose was the same as that of seedlings in the CK2 group, Therefore, 5, 10, and 30 mM trehalose could not promote the growth of watermelon seedlings under salt stress. However, 20 mM trehalose significantly promoted the growth of watermelon seedlings under salt stress (Figure 1A). Overall, 20 mM trehalose treatment significantly increased the stem diameter (Figure 1B), biomass (Figure 1C), and plant height of watermelon seedlings (Figure 1D); significantly reduced MDA content (Figure 1E) of roots; and significantly increased activities of enzymes, such as POD (Figure 1F), SOD (Figure 1G), and CAT (Figure 1H), in watermelon roots. In addition, 20 mM trehalose significantly increased K+/Na+ ratio (Figure 1I). These results indicated that a certain concentration of exogenous trehalose can improve the salt tolerance of watermelon.

### 3.2. RNA-seq Analysis after Trehalose Treatment to Watermelon Seedlings under Salt Stress

To explore the molecular mechanism underlying the improvement in salt tolerance of watermelon by exogenous trehalose, RNA sequencing was performed using the roots of watermelon seedlings, with three biological replicates for each group (a total of nine samples). After data filtering, each sample produced 6.46 Gb data on average. The data quality Q30 was greater than 94.28%; the average genome alignment rate was 92.11%, and the average gene set alignment rate was 63.30% (Supplementary Table S2). In general, the transcriptomic data were of reliable quality and could be used for subsequent analysis.

**Figure 2:**
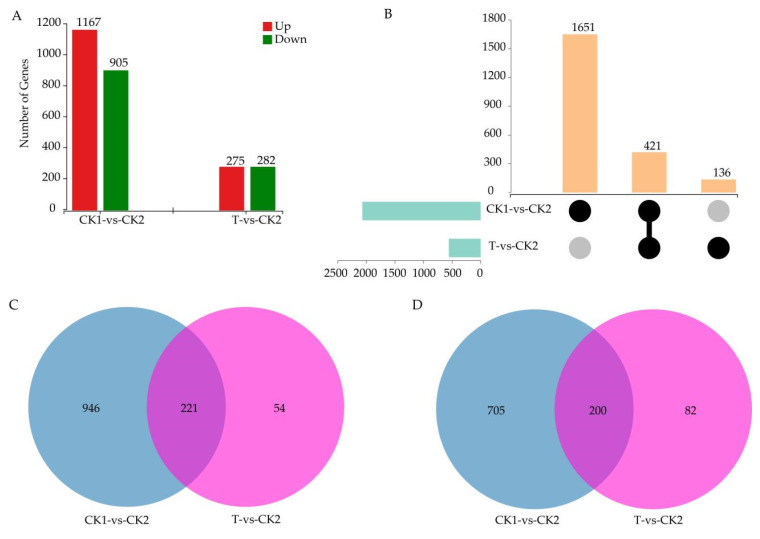
Statistics for DEGs. (**A**) The number of up- and down-regulated DEGs in the root. (**B**) The number of up- and down-regulated DEGs in the leaf. (**C**) Venn diagrams of DEGs for comparison groups in the root. (**D**) Venn diagrams of DEGs for comparison groups in the leaf. CK1 represents watermelon seedlings cultured in normal Hoagland solution; CK2 represents watermelon seedlings cultured in Hoagland solution containing 150 mM NaCl, and T represents watermelon seedlings cultured in Hoagland solution containing 150 mM NaCl and 20 mM trehalose.

A total of 20,128 genes were detected in all samples, including 19,738 known genes and 390 predicted new genes. Overall, 2629 differentially expressed genes (DEGs) were obtained in the two comparison groups (CK1–CK2 and T–CK2). The number of DEGs in CK1–CK2 (2072) was significantly higher than that in T–CK2 (557) (Figure 2A). In the two comparison groups, there were 421 shared DEGs (Figure 2B). Among them, 221 DEGs were upregulated (Figure 2C), and 200 were downregulated (Figure 2D). These results indicated that a large number of genes changed dramatically at the transcription level when watermelon seedlings were subjected to salt stress, and trehalose alleviated the damage to watermelon due to salt stress to a great extent.

### 3.3. Verification of DEGs Using qPCR

**Figure 3:**
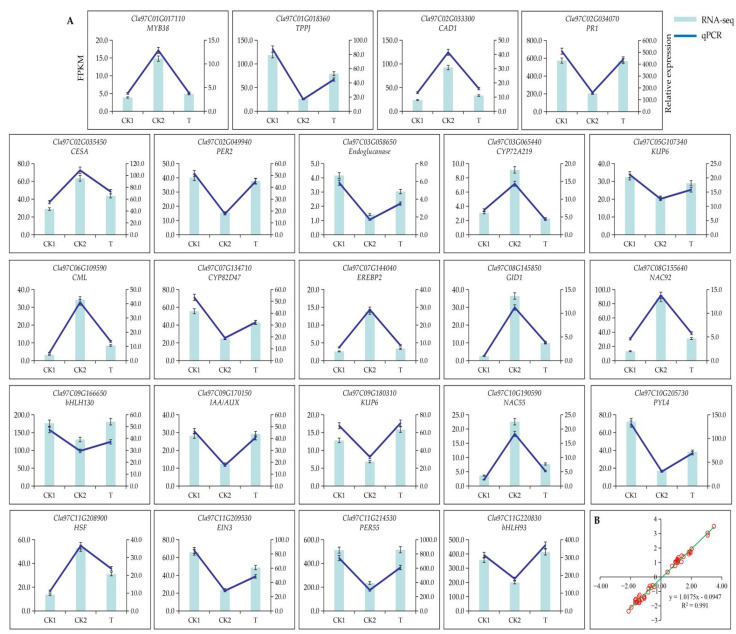
Transcriptomic data were validated using qPCR. (**A**) Gene expression patterns of RNA-seq and qPCR. (**B**) The consistency of RNA-seq and qPCR data was demonstrated based on scatter plot. X axis represents log_2_(CK2/CK1) and log_2_(T/CK2) of RNA-seq, Y axis represents log_2_(CK2/CK1) and log_2_(T/CK2) of qPCR.

To verify the reliability of transcriptomic data, 23 DEGs were randomly selected for qPCR verification (Figure 3). The results revealed that the gene expression pattern of RNA-seq data was highly consistent with qPCR data (Figure 3A, Supplementary Table S3), with the correlation as high as 0.9911 (Figure 3B), confirming the reliability of transcriptomic data.

### 3.4. GO Classification and KEGG Pathway Enrichment Analysis of DEGs

**Figure 4:**
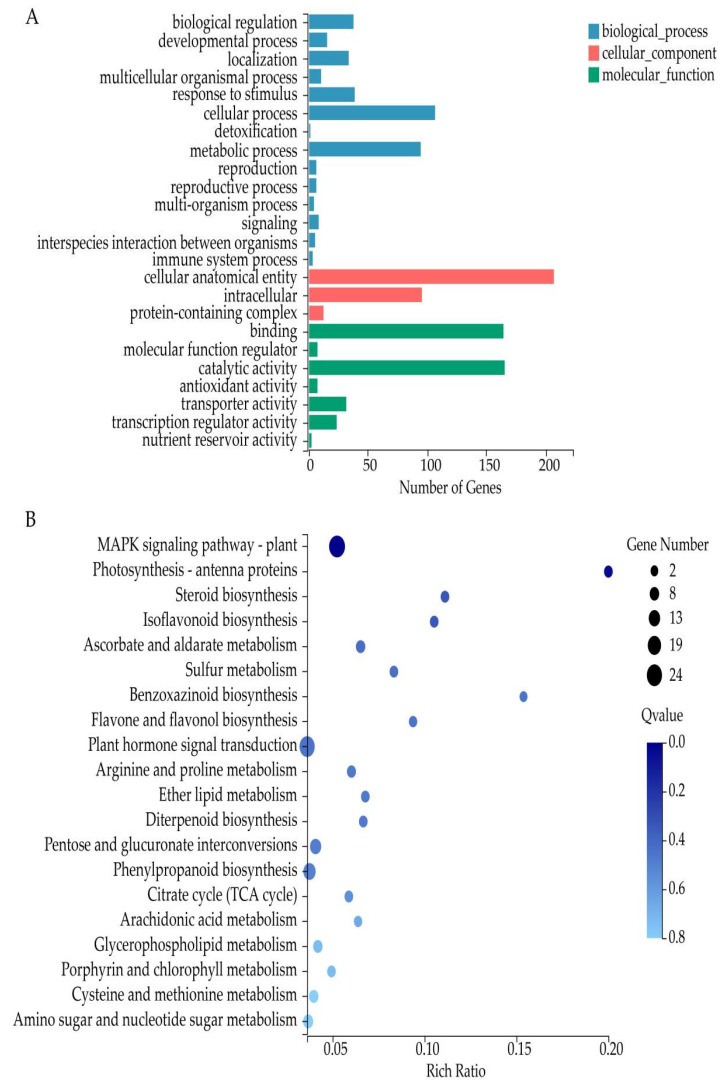
GO classification and KEGG pathway enrichment analysis of DEGs. (**A**) GO classification of DEGs. (**B**) KEGG pathway enrichment analysis of DEGs.

To analyze the main response pathways to salt stress in watermelon seedlings, the shared DEGs were mapped to GO database. They were classified on the basis of biological process, cell component, and molecular function (Figure 4A). To be specific, 421 DEGs were classified into 24 GO terms, including 14 terms of biological process (among which cellular process (105) and metabolic process (93) contained the highest number of genes), 3 terms of cell component (among which cellular anatomical entity contained the highest number of genes (204)), and 7 terms of molecular function (of which catalytic activity (163) and binding (162) contained the highest number of genes; Figure 4A). Furthermore, we performed KEGG enrichment to analyze the functions of shared DEGs and 20 top enriched metabolic pathways were selected (Figure 4B). The main enriched pathways were MAPK signaling pathway involving in plant hormone signal transduction, phenylpropanoid biosynthesis, pentose and glucuronate interconversions, and amino sugar and nucleotide sugar metabolism. MAPK signaling pathway—plant (ko04016, 24 DEGs), plant hormone signal transduction (ko04075, 22 DEGs), and phenylpropanoid biosynthesis (ko00940, 14 DEGs) had the highest number of genes (Figure 4B). These results suggested that these DEGs were activated by various molecular mechanisms, and these pathways may play an important role in the response of watermelon seedlings to salt stress under trehalose treatment.

### 3.5. TFs Responding to Trehalose Treatment under Salt Stress

**Figure 5:**
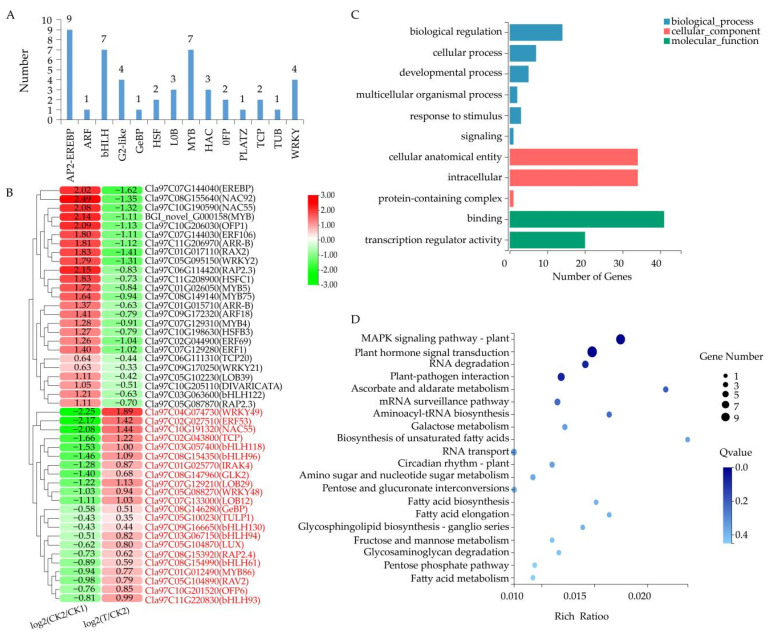
Analysis of differentially expressed TFs. (**A**) Classification analysis of TFs. (**B**) Analysis of expression levels of TF encoding genes. The color scale ranges from −3 to 3, with green indicating downregulation and red indicating upregulation (see the color set scale). (**C**) GO classification of TFs. (**D**) KEGG pathway enrichment analysis of TFs. CK1 represents watermelon seedlings cultured in normal Hoagland solution; CK2 represents watermelon seedlings cultured in Hoagland solution containing 150 mm NaCl, and T represents watermelon seedlings cultured in Hoagland solution containing 150 mM NaCl and 20 mM trehalose.

We identified 47 TFs from shared DEGs between the two comparison groups (Figure 5). They were grouped into 14 subfamilies according to their protein domains, including AP2-EREBP, ARF, bHLH, G2-like, GeBP, HSF, LOB, MYB, NAC, OFP, PLATZ, TCP, TUB, and WRKY. Genes encoding TFs AP2-EREBP (nine DEGs), bHLH (seven DEGs), and MYB (seven DEGs) were the most abundant in these subfamilies (Figure 5A). Furthermore, more than 46.8% TFs (22 DEGs; gene ID marked in red) (Figure 5B) were significantly upregulated in the comparison group T–CK2. It is worth noting that the number of genes encoding bHLH TF was the highest (6 DEGs). We further performed GO annotation and KEGG enrichment pathway analysis to better understand the functions of these 47 TFs. The results revealed that they were divided into 11 GO terms. Binding (GO:0005488) contained the highest number of TF coding genes (41) (Figure 5C), which was highly consistent with the basic function of TF, that is, regulating the transcription of downstream genes by binding to the promoter region of genes. For KEGG enrichment pathway analysis (Figure 5D), MAPK signaling pathway—plant (ko04016) and plant hormone signal transduction (ko04075) contained the greatest number of TF encoding genes (8 and 11 DEGs, respectively; Supplementary Table S4. Moreover, in these two pathways, bHLH TF encoding genes were the most in number, containing six DEGs (Cla97C03G067150, Cla97C03G063600, Cla97C08G154350, Cla97C08G154990, Cla97C09G172230, and Cla97C11G220830). These results suggested that TFs, especially bHLH, may involve in regulating salt tolerance in watermelon seedlings in response to trehalose treatment.

### 3.6. Analysis of Key KEGG Enrichment Pathways for DEGs

**Figure 6:**
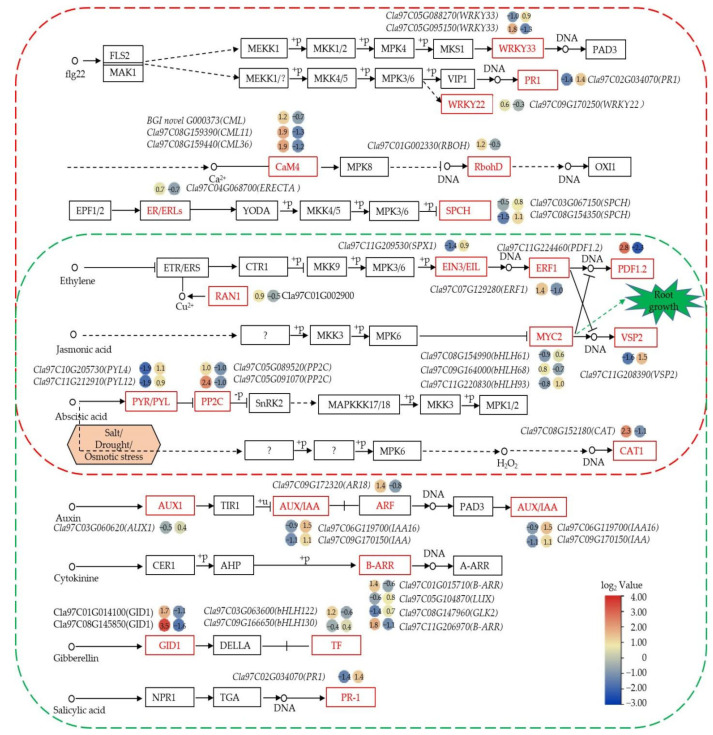
Analysis of MAPK signaling pathway—plant and plant hormone signal transduction in terms of DEGs. Red dotted box represents analysis of MAPK signaling pathway in terms of DEGs. Green dotted box represents analysis of plant hormone signal transduction in terms of DEGs. Genes in red line frame represent DEGs. The dots on the left represent the CK2–CK1 comparison group, and those on the right represent the T–CK2 comparison group. The color scale ranges from −3 to 4, with blue indicating down-regulation and red indicating up-regulation (see the color set scale). CK1 represents watermelon seedlings cultured in normal Hoagland solution; CK2 represents watermelon seedlings cultured in Hoagland solution containing 150 mM NaCl, and T represents watermelon seedlings cultured in Hoagland solution containing 150 mM NaCl and 20 mM trehalose.

**Figure 7:**
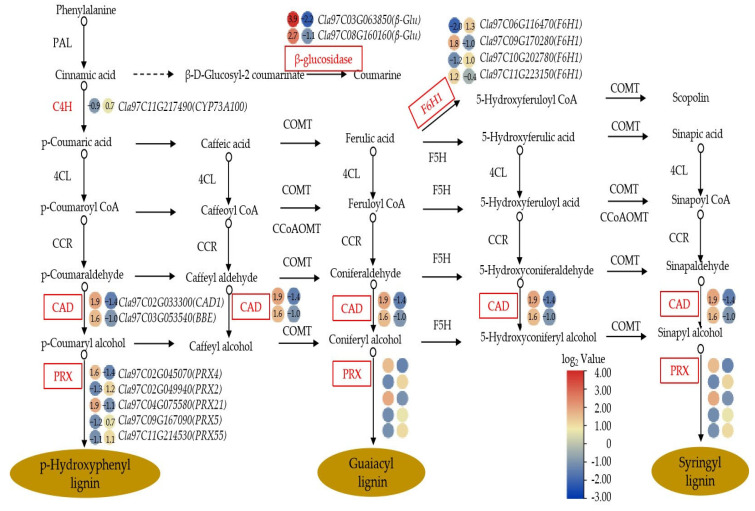
Analysis of phenylpropanoid biosynthesis in terms of DEGs. The dots on the left represent the CK2–CK1 comparison group, and those on the right represent the T–CK2 comparison group. The color scale ranges from −3 to 4, with blue indicating down-regulation and red indicating up-regulation (see the color set scale). CK1 represents watermelon seedlings cultured in normal Hoagland solution; CK2 represents watermelon seedlings cultured in Hoagland solution containing 150 mM NaCl, and T represents watermelon seedlings cultured in Hoagland solution containing 150 mM NaCl and 20 mM trehalose.

We further analyzed the DEGs that enriched in MAPK signaling pathway—plant, plant hormone signal transduction and phenylpropanoid biosynthesis. For example, MAPK signaling pathway—plant involved six biological processes, in which 10 DEGs were significantly up-regulated and 14 DEGs were significantly down regulated after trehalose treatment; plant hormone signal transduction involved seven plant hormone signaling pathways, in which 12 DEGs were significantly up-regulated and DEGs were are significantly down regulated after trehalose treatment (Figure 6). In addition, MYC2 of jasmonic acid (JA) signaling pathway played a role in regulating root growth; *Cla97C08G154990* and *Cla97C11G220830* of the *MYC2* were significantly up-regulated by trehalose treatment. Moreover, abscisic acid (ABA) can respond to salt, drought, and osmotic stress in plants; *Cla97C10G205730* and *Cla97C11G212910* were also significantly up-regulated after trehalose treatment. For phenylpropanoid biosynthesis, 14 DEGs were involved in the biosynthesis of three types of lignin, with up-regulation of 6 DEGs and significant down-regulation of 8 DEGs after trehalose treatment (Figure 7). These genes included one C4H (*Cla97C11G217490*), two β-glucosidases (*Cla97C03G063850* and *Cla97C08G160160*), four F6H1s (*Cla97C06G116470, Cla97C09G170280, Cla97C10G202780* and *Cla97C11G223150*), two CADs (*Cla97C02G033300* and *Cla97C03G053540*), and five PERs (*Cla97C02G045070, Cla97C02G049940, Cla97C04G075580, Cla97C09G167090* and *Cla97C11G214530*). These results suggested that trehalose may improve salt tolerance of watermelon seedlings by changing the expression level of genes related to plant hormones and lignin.

### 3.7. Analysis of Salt-Stress Related DEGs in Response to Trehalose Treatment

**Figure 8:**
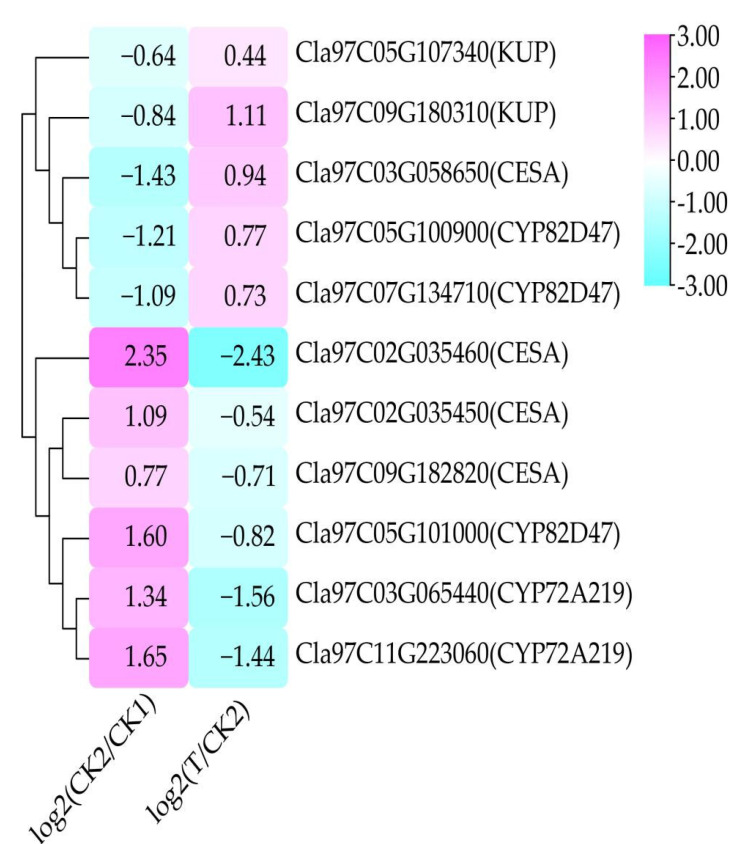
Analysis of DEGs responding to trehalose treatment under salt stress. The color scale ranges from −3 to 3, with cyan indicating down-regulated and pink indicating up-regulated (see the color set scale). CK1 represents watermelon seedlings cultured in normal Hoagland solution; CK2 represents watermelon seedlings cultured in Hoagland solution containing 150 mM NaCl, and T represents watermelon seedlings cultured in Hoagland solution containing 150 mM NaCl and 20 mM trehalose.

Among the 421 DEGs, some salt-responsive genes include 2 *KUPs* (KUP system potassium uptake protein), 4 *CESAs* (cellulose synthase A), and 5 *CYPs* (cytochrome P450) (Figure 8). Among them, two *KUPs* (*Cla97C05G107340* and *Cla97C09G180310*), one *CESA* (*Cla97C03G058650*), and two *CYPs* (*Cla97C05G100900* and *Cla97C07G134710*) were down-regulated under salt stress, whereas they were up-regulated under trehalose treatment. However, three *CESAs* (*Cla97C02G035450*, *Cla97C02G035460*, and *Cla97C09G182820*) and three *CYPs* (*Cla97C05G101000*, *Cla97C03G065440*, and *Cla97C11G223060*) were up-regulated under salt stress and were down-regulated under trehalose treatment. The results revealed that these salt-responsive genes were non-redundant, and trehalose might improve the salt tolerance of watermelon seedlings by dynamically regulating the expression of genes.

### 3.8. DEM Analysis of Trehalose Treatment in Response to Salt Stress

**Figure 9:**
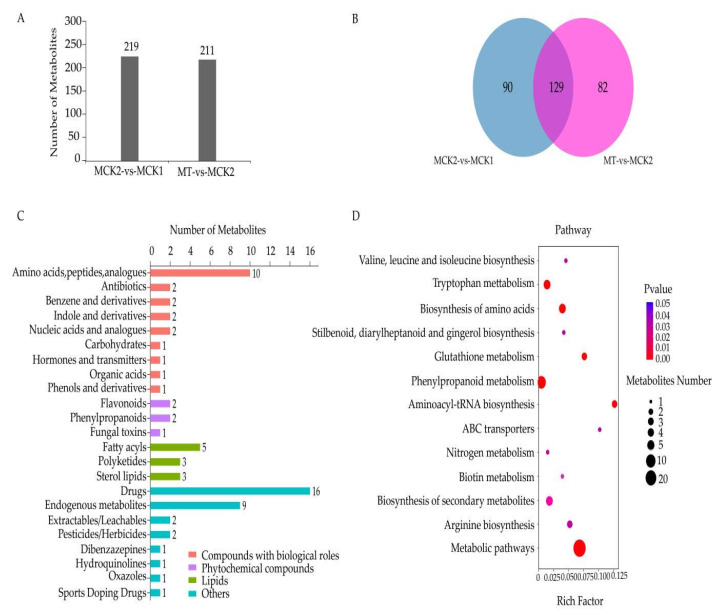
Statistics for DEMs. (**A**) The number of DEMs in the root. (**B**) Venn diagrams of DEMs in the comparison groups in the root. (**C**) KEGG categories of DEMs. (**D**) KEGG pathway enrichment analysis of DEMs. MCK1 represents metabolites of watermelon seedlings cultured in normal Hoagland solution; MCK2 represents metabolites of watermelon seedlings cultured in Hoagland solution containing 150 mm NaCl, and MT represents metabolites of watermelon seedlings cultured in Hoagland solution containing 150 mM NaCl and 20 mM trehalose.

We performed metabolic profiling using watermelon root samples (the samples are identical to those used for RNA-seq) to assess the metabolic effects of trehalose treatment on the root system of watermelon seedlings under salt stress (Figure 9). Compared with MCK1 treatment, 219 differentially expressed metabolites (DEMs) were detected, among which 114 were up-regulated and 105 were down-regulated. Between MCK2 and MT treatments, 211 DEMs were detected, 105 up-regulated and 106 down-regulated compared with MCK2 treatment (Figure 9A) (Supplementary Table S5).

A total of 129 shared DEMs were screened in the two comparison groups, and their KEGG categories and KEGG enrichment pathways were analyzed (Figure 9B). The results revealed that 72 of 129 DEMS were successfully classified into four groups, including compounds with biological roles (22 DEMs), phytochemical compounds (5 DEMs), lipids (11 DEMs), and others (34 DEMs; Figure 9C). In addition, 10 “amino acids, peptides, and analogues” (L-tryptophan, D-(-)-glutamine, L-glutamic acid, 3-methylhippuric acid, L-(+)-citrulline, L-pyroglutamic acid, L-isoleucine, D-glutamic acid, biocytin, and N-isovalerylglycine) in compounds with biological roles, 2 “flavonoids” (ononin and liguiritigenin-7-O-β-D-apiosyl-4’-O-β-D-glucoside) and 2 “phenylpropanoids” (4,5-dicaffeoylquinic acid and trans-cinnamic acid) in phytochemical compounds, and 5 “fatty acyls” (13(S)-HOTrE, 15-keto prostaglandin E1, 10-nitrolinoleate, docosahexaenoic acid, and 2-trans-hexadecenoic acid) in lipids were the most in number (Supplementary Table S5). Moreover, 19 of 129 DEMs were enriched in 13 KEGG pathways (Figure 9D, Supplementary Table S6); metabolic pathways (map01100) and phenylpropanoid biosynthesis (map00940) contained the most DEMs, 15 (UDP-N-acetylglucosamine, estriol, L-isoleucine, biocytin, trans-cinnamic acid, L-(+)-citrulline, D-(-)-glutamine, L-pyroglutamic acid, D-glutamic acid, L-glutamic acid, L-tryptophan, N-acetylserotonin, cinnamic acid, melatonin, and cytidine) and 4 (trans-cinnamic acid, L-tyrosine, L-phenylalanine, and cinnamic acid), respectively.

### 3.9. Combined Analysis of DEGs and DEMs

**Figure 10:**
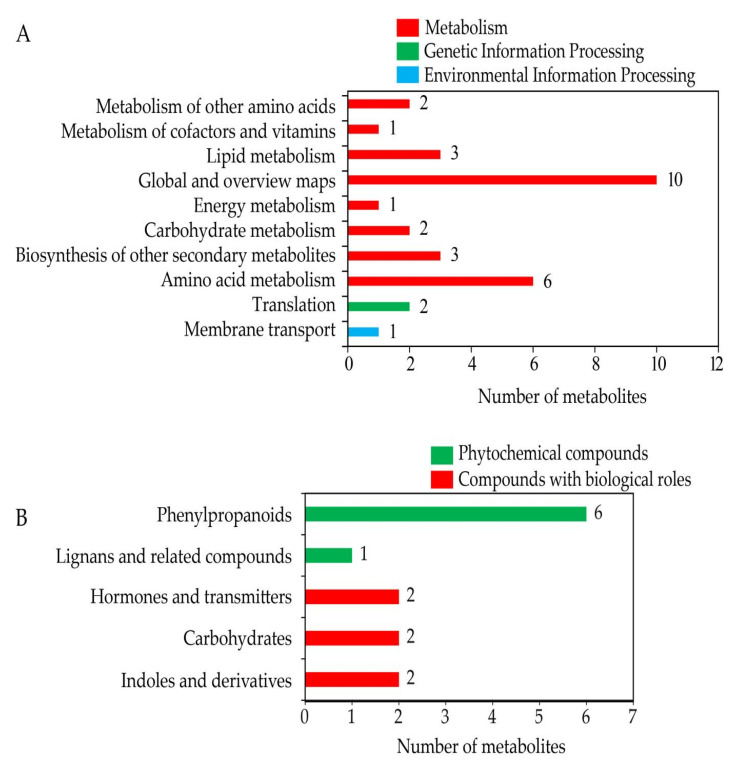
Combined analysis of transcriptomic and metabolomic data. (**A**) Classification of combined DEMs. (**B**) KEGG categories of combined DEMs.

To further understand the relationship between proteomic and transcriptomic data, a combined analysis of 421 DEGs and 129 DEMs was conducted (Supplementary Table S7). As a result, a total of 14 metabolites were successfully screened and divided into three major categories. Among them, metabolism included eight categories, and genetic information processing and environmental information processing included one category each (Figure 10A, Supplementary Table S8). Furthermore, 13 of 14 DEMs were divided into two major KEGG categories. Among them, phytochemical compounds contained two categories, and compounds with biological roles contained three categories (Figure 10B, Supplementary Table S9), with L-tyrosine, L-phenylalanine, cinnamic acid, trans-cinnamic acid, L-tryptophan and 4-Methoxycinnamaldehyde in “phenylpropanoids”; tracheloside in “lignans and related compounds”; salicylic acid (SA) and JA in “hormones and transmitters”; UDP-N-acetylglucosamine and L-glutamic acid in “carbohydrates”; and melatonin and vindoline in “indoles and derivatives.” The results indicated that trehalose might improve the tolerance of watermelon seedlings to salt stress by changing the levels of these metabolites.

### 3.10. Correlation and Co-Expression Network Analysis of Candidate Genes and Transcription Factors

**Figure 11:**
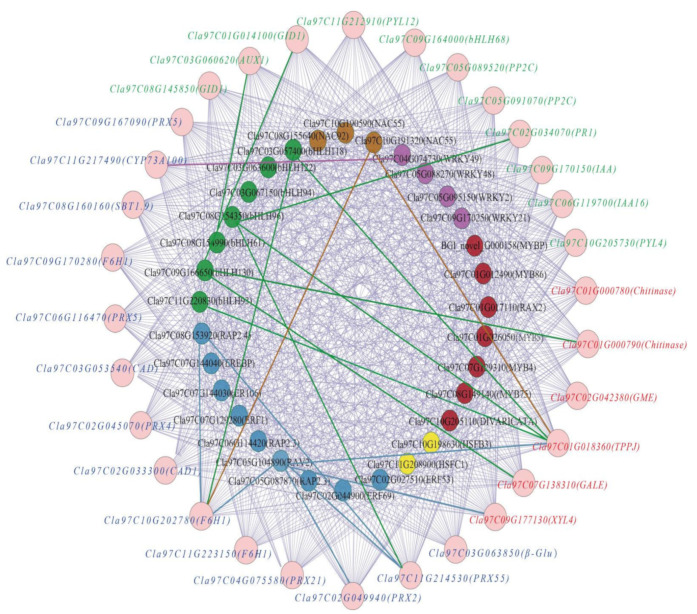
Co-expression network analysis of TFs and a series of DEGs with correlation coefficient > 0.9. The internal green, blue, yellow, red, purple, and brown dots represent bHLH TFs, AP2-EREBP TFs, HSF TFs, MYB TFs, WRKY TFs, and NAC TFs, respectively. Lines marked with different colors represent TFs that are up-regulated, and the color of the line is represented by the color of the corresponding TF. The external red, green, and blue fonts represent sugar-related DEGs, plant-hormone-related DEGs, and lignin-related DEGs, respectively.

To further screen the key TFs that may regulate the genes, we analyzed the relationship between 32 salt-tolerance-related TFs (from Figure 5) and 31 DEGs. The 32 TFs included 9 AP-EREBPs, 7 bHLHs, 2 HSFs, 7 MYBs, 3 NACs, and 4 WRKYs. The 31 DEGs included 14 lignin-related DEGs, 11 plant-hormone-related DEGs, and 6 carbohydrate-related DEGs (Supplementary Figure S1). In addition, most TFs and DEGs exhibited a high correlation. Further, we constructed a co-expression network with 171 network lines using data with correlation coefficients > 0.9, containing 28 TFs and 31 DEGs (Figure 11, Supplementary Table S10). Of 28 TFs, 9 TFs were up-regulated by trehalose treatment (Figure 5), and there were 11 corresponding genes. Among nine TFs, there were five bHLH TFs (Cla97C11G220830, Cla97C03G057400, Cla97C08G154350, Cla97C08G154990, and Cla97C09G166650). In addition, TFs corresponding to genes associated with trehalose biosynthesis (Cla97C01G018360) were the most in number (Cla97C11G220830, Cla97C05G104890, Cla97C03G057400, Cla97C08G154350, and Cla97C10G191320). The correlation between Cla97C11G220830 and Cla97C01G018360 was the highest (0.992). Moreover, lignin-biosynthesis-related gene *F6H1* (*Cla97C10G202780*) corresponded to four TFs (Cla97C05G104890, Cla97C08G153920, Cla97C03G057400, and Cla97C10G191320), and *PER* (*Cla97C11G214530*) corresponded to three TFs (Cla97C05G104890, Cla97C08G153920, and Cla97C08G154350) (Supplementary Table S11). These results indicated that lignin plays a major role in salt tolerance, and potential TFs, especially bHLH TF Cla97C11G220830, regulating lignin biosynthesis and trehalose-biosynthesis-related genes may play crucial roles in improving salt tolerance of watermelon.

## 4. Discussion

In this study, 20 mM of exogenous trehalose greatly improved the tolerance of watermelon seedlings to salt stress. This may be because trehalose can protect membrane system, reduce the degree of damage to cell membrane, and maintain ion balance by increasing the activities of related enzymes (SOD, POD, and CAT) and ratio of K^+^/Na^+^ and decreasing MDA content (Figure 1). This is consistent with previous studies, indicating that appropriate concentration of trehalose can improve stress resistance of plants by maintaining cell membrane, protein functions, and ionic balance [32,39,40]. Mostofa et al. [41] reported that pretreatment of rice with 10 mM trehalose for 2 days can effectively inhibit the production of ROS and MDA and significantly reduce the damage caused by salt stress; even CAT, GST, GPX, and GR activities in rice seedlings could be maintained under salt stress. Wang et al. [42] reported that 0.3 mM trehalose was the optimal concentration that could significantly improve drought tolerance, whereas trehalose at 0.5 mM aggravated the damage due to drought stress on sweet sorghum seedlings.

The alleviation of the damage caused by salt stress in watermelon seedlings by trehalose treatment was mainly reflected in significantly lower DEGs in T–CK2/CK2–CK1 (Figure 2) and significantly lower DEMs in the comparison groups MT–MCK2/MCK2–MCK1 (Figure 9). At the same time, some DEGs responding to trehalose treatment under salt stress were detected, such as *CESAs*, *KUPs,* and *CYPs* (Figure 8). The cell wall is the first line of defense against salt stress in plants [42]. When plant roots are subjected to external salt stress, the cell wall sensing system is the first to receive the salt stress signal [43,44]. Cellulose is a major component of the cell wall and is synthesized by cellulose synthases (CESAs) guided by microtubules on the plasma membrane [45]. Cellulose content in plants decreases under salt stress [46]. In this study, 1 *CESA* (*Cla97C03G058650*) was up-regulated under trehalose treatment, indicating that trehalose may improve the defense of the cell wall by increasing the activity of CESA, thereby improving the tolerance of watermelon seedlings to salt stress. Under salt stress, high concentration of Na^+^ competes with K^+^ uptake by plants, leading to K^+^ leakage through outward rectifying K^+^ channels, reducing the K^+^/Na^+^ ratio, and resulting in Na^+^ poisoning in plants [47,48,49]. Therefore, increasing K^+^ uptake is one of the main strategies for plants to tolerate salt stress [47,50]. There are many HAK/KUP/KT transporters in plants that play an important role in the uptake and transport of K^+^ and regulation of salt tolerance [51]. The overexpression of *AtKUP2* can enhance the salt tolerance of *Arabidopsis thaliana* and accumulate less Na^+^ and more K^+^ in stems and roots compared with wild-type plants [52]. Two *KUPs* (*Cla97C05G107340* and *Cla97C09G180310*) were up-regulated under trehalose treatment, which might play a role in improving K^+^/Na^+^ ratio. Cytochrome P450 is the largest family of enzyme proteins in land plants and plays an important role in defense against adversity [53]. The expression of *CYP82D* in cucumber seedlings under salt stress was significantly up-regulated after brassinolide treatment, which may have a positive regulatory effect on salt tolerance in cucumbetr [54]. Similar results were obtained in this study, with two *CYP82Ds* (*Cla97C05G100900* and *Cla97C07G134710*) significantly up-regulated after trehalose treatment.

SA is an important signaling molecule in the physiological mechanism of plant stress tolerance. Relevant studies have reported that the application of exogenous SA can improve the salt tolerance of plants [55,56,57,58,59]. Moreover, previous studies revealed that the application of exogenous SA and JA significantly enhanced the accumulation of trehalose in watermelon, which in turn restored cell growth under salt stress, and maintained pH, ROS homeostasis, and the integrity of the microtubule skeleton [60]. Furthermore, many studies have reported that JA is involved in the regulation of many plant growth processes, and the regulation of JA on these biological processes often requires synergistic action with other plant hormones to balance the biochemical reactions of plants and their adaptation to the external environment through synergistic or combined action between hormones [61,62,63]. In this study, DEGs and DEMs of SA and JA signaling transduction pathway significantly changed after trehalose treatment (Figure 6, Figure 7, Figure 8, Figure 9 and Figure 10), suggesting that MYC2 TF (Cla97C08G154990, Cla97C09G164000 and Cla97C11G220830) of JA pathway and genes (*PDF1.2*, *Cla97C11G224460*) related to ethylene pathway may jointly regulate salt tolerance (Figure 6).

The lignification degree of plant root cell walls is increased under salt stress, which can effectively prevent intracellular ion absorption and improve the salt tolerance of plants [64]. Salt stress alters many enzymes (shcu as C4H, CAD, and PRX) involved in lignin biosynthesis by changing their expression patterns, regulating lignin synthesis in response to salt stress [65]. For example, overexpression of *PRX* enhanced the salt stress tolerance of transgenic plants [66,67]. In this study, we obtained 5 differentially expressed *PRXs* (Figure 7). In total, 3 (*Cla97C02G049940, Cla97C09G167090, Cla97C11G214530*) of them were significantly up-regulated under trehalose treatment, which may play a role in improving the salt tolerance of watermelon seedlings. Besides PRX, we identified 1 key metabolite, namely, cinnamic acid, in DEMs (which was upregulated in lignin synthesis pathway) and 1 upregulated gene, namely, *Cla11G217490* encoding cinnamate-4-hydroxylase (C4H) in DEGs (which directly catalyzes cinnamic acid). Studies have reported that C4H is one of the major flux-controlling enzymes in plant lignification. The expression and activity of C4H can directly affect lignin production [68,69]. The synthesis process of lignin requires the coordinated participation of multiple enzyme genes and is also regulated by the combination of TFs–gene network [70]. At present, AP2, bHLH, MYB, NAC, and WRKY TFs have been verified to participate in the regulation of lignin synthesis [65,71,72,73]. In this study, some AP2, bHLH, NAC, and WRKY TFs were highly correlated with the expression patterns of genes related to lignin synthesis (Figure 11), suggesting that these TFs may also be involved in lignin synthesis and play a role in regulating the salt tolerance of watermelon seedlings.

In addition to trehalose, other soluble sugars such as glucose, sucrose, and fructose also act as osmoprotectants, providing membrane protection and scavenging excess ROS to effectively resist salt stress [74]. In our metabolomic data, β-estradiol-17β-glucuronide, liguiritigenin-7-O-β-D-apiosyl-4′-O-β-D-glucoside, and UDP-N-acetylglucosamine were found in the process of sugar metabolism, and some DEGs of sugar metabolism were also detected in transcriptomic analysis (Supplementary Tables S5 and S10). Among the DEGs, *Cla97C01G018360* was a gene that encoded TPP. In *A.thaliana*, overexpression of *AtTPPD* improved salt tolerance of transgenic plants [75]. In addition, overexpression of *TaTPP7* in *A.thaliana* increased trehalose content in transgenic plants and improved salt tolerance in A.*thaliana*. In rice, plants overexpressing *OsTPP1* accumulated high concentrations of trehalose and exhibited a high tolerance to salt [76]. Interestingly, among all the correlation data, the correlation coefficient between bHLH TF Cla97C11G220830 and *Cla97C01G018360* was the highest, suggesting that Cla97C11G220830 may play a role in regulating *Cla97C01G018360* expression. In addition, bHLH regulates the expression of downstream genes by binding to *cis*-acting elements in the promoter region of salt-tolerant genes and is widely involved in response to salt stress. In *A. thaliana*, AtbHLH122 can directly bind to E-Box/G-box in *CYP707A3* promoter and inhibit its expression, thus significantly increasing ABA level in cells; moreover, overexpressing *AtbHLH122* increased tolerance to NaCl stress [77]. In rice, OrbHLH001 protein can specifically bind to E-box of *OsAKT1* promoter region and induce *OsAKT1* expression to regulate Na^+^/K^+^ ratio, significantly improving tolerance of rice to salt stress [78]. Analysis of the promoter region of *Cla97C01G018360* revealed that there were 1 E-box and 2 G-boxes (Supplementary Table S12). It was speculated that Cla97C11G220830 could bind with E-box/G-box of *Cla97C01G018360* to directly regulate its expression, leading to promote trehalose accumulation and improving salt tolerance of watermelon seedlings.

**Figure 12:**
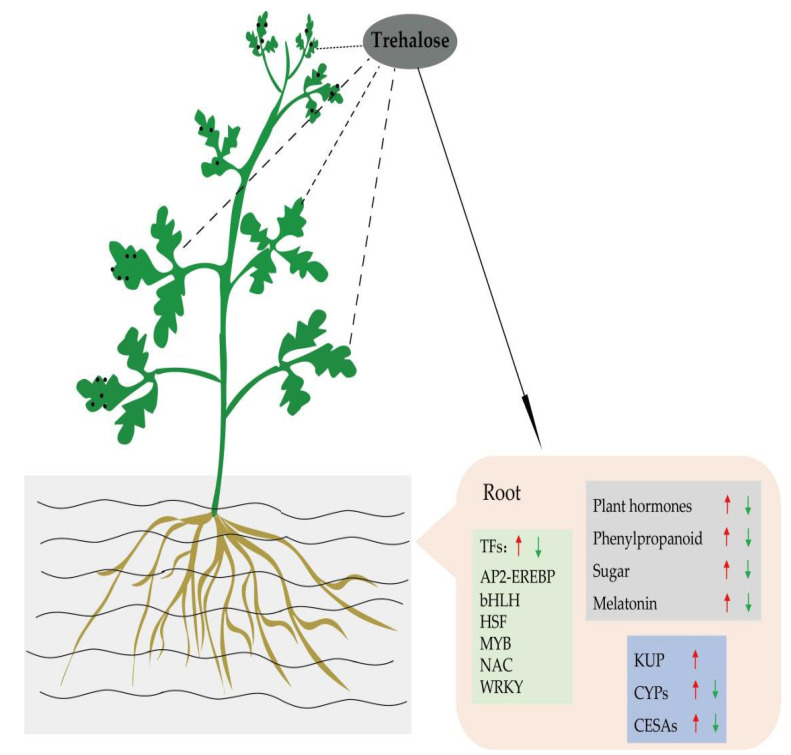
Hypothesized mechanism of exogenous trehalose enhancing salt tolerance of watermelon seedlings.

Based on the above results, we constructed a hypothesized network of regulation of salt tolerance in watermelon seedlings by trehalose (Figure 12). The genes encoding TFs (AP2-EREBP, bHLH, HSF, MYB, NAC, and WRKY); related genes in watermelon such as KUP, CPYs, and CESAs; and the genes and metabolites related to signal transduction for plant hormones, phenylpropanoid biosynthesis, and sugar metabolism pathway together improved the salt tolerance of watermelon seedlings.

## 5. Conclusions

In this study, the effects of various concentrations of exogenous trehalose on watermelon seedlings under salt stress were investigated. The changes in transcriptomic and metabolomic profiles were comprehensively analyzed in the roots of watermelon seedlings treated with trehalose under salt stress. The results revealed that 20 mM exogenous trehalose could significantly improve the salt tolerance of watermelon seedlings and promote the expression of salt-tolerance-related TFs and genes. Moreover, gene expression and metabolite accumulation in phenylpropanoid biosynthesis, plant hormone signal transduction, and carbohydrate biosynthesis pathways enhanced salt tolerance. These findings will provide specific direction for further understanding the mechanism of salt stress tolerance by watermelon with trehalose and provide a scientific theoretical basis for the practical application of trehalose in the future.

## Data Availability

The datasets for this study can be found in the National Center for Biotechnology Information (NCBI) repository, bioproject: PRJNA836010.

## Supplementary Materials

The following supporting information can be downloaded at: https://www.mdpi.com/article/10.3390/cells11152338/s1, Figure S1: Correlation analysis of TFs and a series of DEGs. Table S1: Primers for q-PCR.; Table S2: Basic information about transcriptomic data; Table S3: Cycle threshold of qPCR of DEGs; Table S4: TFs involved in MAPK signaling pathway—plant and plant hormone signal transduction. Table S5: DEMs of MCK2-MCK1 and MT-MCK2; Table S6: KEGG enrichment pathway of shared DEMs; Table S7: Correlation analysis of 421 DEGs and 129 DEMs; Table S8: Classification of DEMs obtained by combined analysis; Table S9: KEGG pathway of DEMs obtained by combined analysis; Table S10: Correlation coefficients greater than 0.9 of TFs and DEGs; Table S11: Up-regulated transcription factors and corresponding genes; Table S12.E-box/G-box of Cla97C01G018360 promoter region.

## Abbreviations

POD/PRX	peroxidase
SOD	superoxide
CAT	catalase
MDA	malondialdehyde
LC-MS	liquid chromatography-mass spectrometry
TF	transcription factor
TPS	trehalose phosphate synthase
TPP	trehalose phosphate phosphatase
APX	ascorbic acid peroxidase
Pro	proline
ROS	reactive oxygen species
ORF	open reading frame
ABA	abscisic acid
KUP	potassium uptake protein
CESA	cellulose synthase A
CYP	cytochrome P450
DEG	differentially expressed gene
DEM	differentially expressed metabolite
SA	salicylic acid
JA	jasmonic acid
