# Supplementary material for: Transcriptomic and Metabolomic Analysis of the Effects of Exogenous Trehalose on Salt Tolerance in Watermelon (Citrullus lanatus)
Source: Cells. 2022 Jul 29;11(15):2338. doi: 10.3390/cells11152338 (PMC9367363; doi:10.3390/cells11152338)
Supplement: Supplementary file 1 [file cells-11-02338-s001.zip › supplementary Figure S1.pdf]

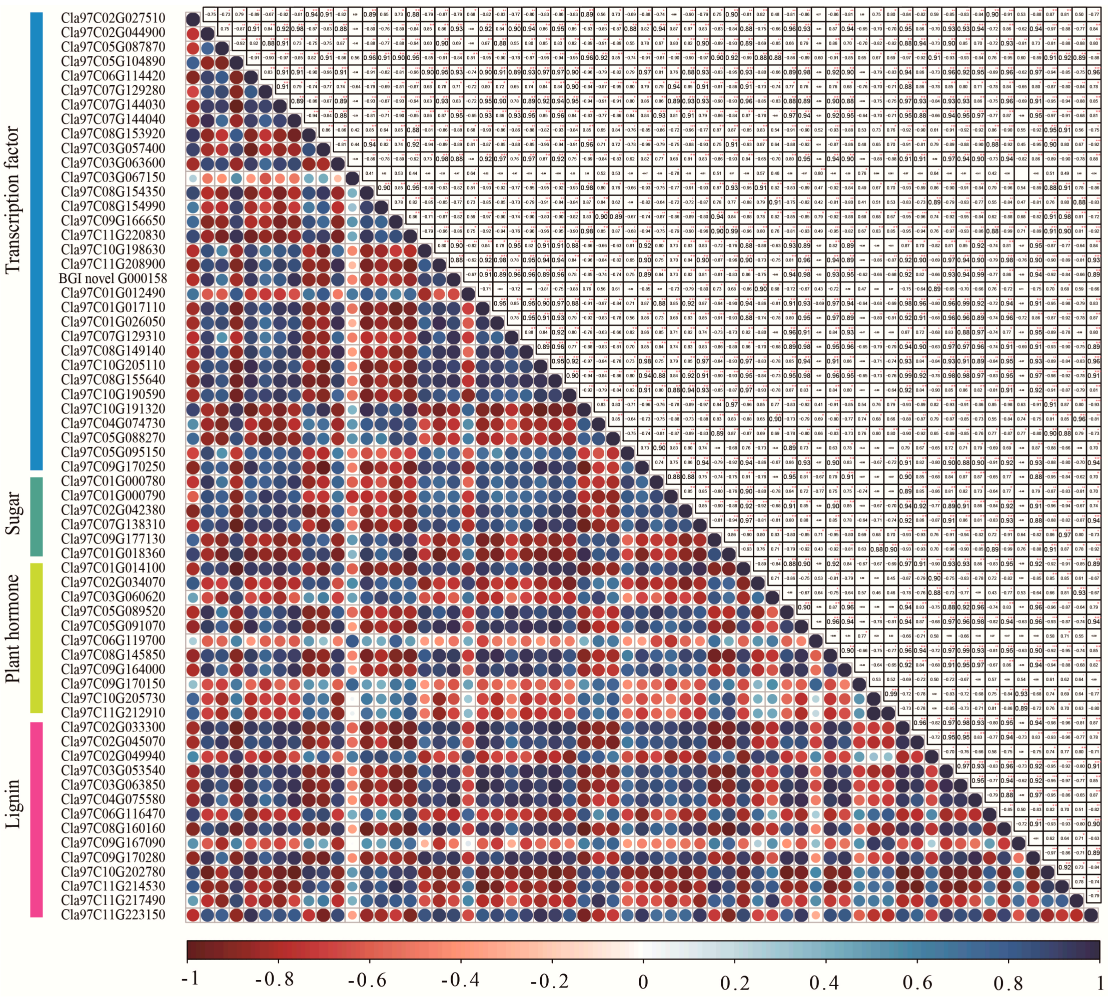

Figure S1. Correlation analysis of TFs and a series of DEGs. The external red, green, and blue fonts represent sugar-related DEGs, plant-hormone-related DEGs, and lignin-related DEGs, respectively.
